# Popliteal Artery Entrapment or Chronic Exertional Compartment Syndrome?

**DOI:** 10.1155/2017/6981047

**Published:** 2017-08-14

**Authors:** Christopher Gaunder, Brandon McKinney, Jessica Rivera

**Affiliations:** San Antonio Military Medical Center (SAMMC), San Antonio, TX, USA

## Abstract

Diagnosis of lower limb pain in an athlete can be a challenging task due to the variety of potential etiologies and ambiguity of presenting symptoms. Five of the most commonly encountered causes of limb pain in athletes are chronic exertional compartment syndrome (CECS), medial tibial stress syndrome (MTSS), tibial stress fractures, soleal sling syndrome, and popliteal artery entrapment syndrome (PAES). Of these, the least frequent but potentially most serious of the pathologies is PAES. With an incidence of less than 1% seen in living subject studies, the condition is rare. However, a missed diagnosis will likely lead to progression of the disease and potential for unnecessary invasive procedures (McAree et al. 2008). In this paper, we present a young athlete misdiagnosed and treated for chronic exertional compartment syndrome. In both descriptive and a quick-reference table format, we review current literature and discuss how best to distinguish functional PAES from other causes of activity-related leg pain.

## 1. Introduction

Popliteal artery entrapment syndrome (PAES) is a condition caused when the popliteal artery becomes compressed by the medial head of the gastrocnemius proximally and fascial band of the soleus distally during activity, leading to painful claudication type symptoms and oftentimes paresthesias. PAES is classically differentiated into two categories: anatomic and functional. Anatomic PAES results from either aberrant anatomy of the proximal gastrocnemius, the popliteal artery, or a combination thereof. This anatomic aberrancy predisposes the artery to compression and is further subclassified into five types based on which anatomic variation is present [1]. Functional PAES is found in patients such as the one below where a classic anatomic variation is not present; rather a hypertrophied gastrocnemius functionally results in a similar mode of compression during exercise [2].

We present a case of a young active male who was misdiagnosed and treated for chronic exertional compartment syndrome and review how best to distinguish functional PAES from other causes of activity-related leg pain.

## 2. Case Report

Our patient is a 25-year-old active duty male who initially reported a five-month history of bilateral leg pain, left greater than right, which occurred frequently with exercise. His pain localized to his calf muscles and was associated with tenseness, cramping, and numbness about his feet. The timing of his pain onset was variable but occurred every time he attempted to run. Resolution of symptoms typically occurred after 20 to 30 minutes of rest. Initial radiographs and bone scan were negative.

When the patient's postexercise compartment pressures were measured utilizing a Stryker intracompartmental pressure monitoring system, elevation was noted from his preexercise baseline. Preexercise baseline values and postexercise values for each compartment in the left lower extremity can be seen in Table 1. The patient's deep posterior compartment of the left leg increased from 34 mmHg (pre) to 66 mmHg (post). Based on these results, the patient underwent elective left leg fasciotomy at an outside institution. Following an uneventful recovery from the surgery, his symptoms persisted for which he sought no further treatment for two years.

**Figure 1:**
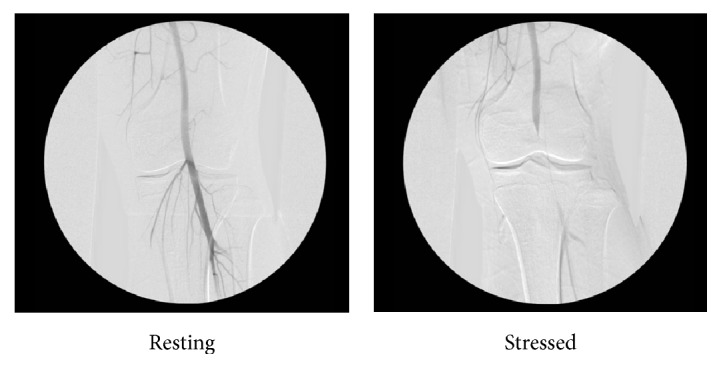
Stress CT angiography, left lower extremity. Notice near complete cessation of flow in the popliteal artery during the stressed or active phase of angiography.

At the time of presentation to our clinic, the patient reported not only a lack of relief following surgery, but a worsening of symptoms in his operative leg. The pain continued to be associated with activity as previously described; however onset of symptoms now occurred with decreased intensity of stimulus. Upon examination of the patient, a decreased posterior tibial pulse which became impalpable during dorsiflexion of the ankle was noted. Furthermore, the patient could immediately reproduce his symptoms through weight bearing plantar flexion. Magnetic resonance imaging (MRI) was obtained and did not indicate anatomic abnormalities of the medial head of the gastrocnemius. An angiogram was then performed by Vascular Surgery service, which detected chronic arterial wall thickening. Stress computed tomography angiography (CTA) of the left lower extremity was performed which demonstrated lack of flow in the popliteal artery during stress (Figure 1). The patient was diagnosed after three years and one unsuccessful surgical procedure with popliteal artery entrapment syndrome.

Following his diagnosis, the patient declined further surgical or invasive interventions. Given the exertional nature of his symptoms, he instead opted to initiate the Medical Evaluation Board (MEB) process with the military. The MEB determines whether or not a military member's medical condition enables him/her to continue to meet medical retention standards in accordance with military regulations. During this review process, he implemented activity modification and continued to follow up in the orthopedic clinic for 6 months. At follow-up, he reported fewer symptomatic events since choosing to discontinue impact and high-intensity activities.

## 3. Discussion

Popliteal artery entrapment syndrome can be difficult to diagnose as the syndrome is relatively rare and the signs and symptoms are very similar to other clinical entities seen in a young, athletic population. Compared to other diagnoses of activity-related leg pain, functional PAES has a low incidence: reports range in incidences of less than 1% in a cohort of military recruits to as high as 3.5% based on postmortem dissections [3, 4]. There are features in our patient's examination and work-up that should have alerted the treating physician to the possibility of PAES.

Patients with PAES, as with our patient, experience pain, cramping, and tenseness in the posterior leg during exertion. Notably our patient reported paresthesias about the sole of his foot which Turnipseed reports as more prognostic of PAES than CECS (40% versus 4.6%) [5]. Neurologic symptoms are variable. However, peroneal nerve dysfunction as might be seen with chronic exertional compartment syndrome or nerve entrapment is not present in PAES [6]. The key examination finding in PAES is weaker distal pulses compared to the uninvolved side or attenuation of the pulses with foot positioned in dorsi- or plantarflexion and knee extension [5–8]. Even in patient without aberrations in proximal gastrocnemius anatomy, this provocative position will cause some proximal compression of the popliteal artery resulting in a positive examination finding and possibly even a reproduction of the patient's leg pain. An ankle-brachial index (ABI) may also be used to aid in the diagnosis as a drop in ABI of 30–50% with ankle dorsiflexion is concerning [5–7].

Various imaging modalities exist in the work-up of PAES. Some institutions have implemented dynamic color duplex ultrasonography (CDUS) as a screening method in all athletes complaining of chronic leg pain during exercise [9]. Others call for the combination of Doppler US and Magnetic Resonance Angiography in all suspected cases [10]. Some institutions report high false-positive rates with use of ultrasound [11–13]. However, in their review of 61 cases of PAES, Corneloup et al. report a specificity of 76% for dynamic CDUS when used only in symptomatic patients with a high threshold (complete cessation of flow in the popliteal artery during dynamic maneuver) [9]. For patients with suspected PAES following CDUS, confirmation with CTA or MR angiography (MRA) is still recommended [9–11, 14]. MRA is preferred for its lack of radiation exposure and detailed soft tissue anatomy. However, some patients have difficulty remaining immobile during the active plantarflexion phase due to MRA's lengthy acquisition time. In contrast, CTA is preferred by some for its accessibility and short acquisition time. While CTA has long been the classical screening and diagnostic tool in functional PAES, newer methods of screening and diagnosis such as dynamic US and MRA have proven their usefulness in recent years and should be considered in the work-up for functional PAES [9–11, 14].

Chronic exertional compartment syndrome is a relatively common condition, occurring in approximately 30% of athletes with chronic leg pain [15]. The pain experienced in this syndrome presents during exercise, typically at a consistent time point following the onset of exercise and often—though not always—resolves once the athlete ceases exertion [16, 17]. The anterior muscle compartment is most often affected; and the condition is most frequently bilateral [16]. Patients will experience pain, cramping, and/or burning and may also exhibit swelling about the affected musculature. Neurologic compromise may also occur, most commonly affecting the peroneal nerve. On physical exam, these patients may have a palpable facial defect allowing muscle herniation. After exercise, the affected compartment will be tender, tense, and painful to passive stretch. Compartment bilateral pressure measurements aid in the diagnosis and should be performed before and after exercise. Resting pressures may be elevated or delayed in returning to normal in this condition; and the diagnosis is typically considered if the pressure is greater than 30 mmHg one minute after ceasing pain provoking exercise [15]. The presence of unilateral compartment pressure increases should stimulate the clinician to investigate the presence of contralateral orthopedic pathology as a source for the unilateral muscle imbalance. It is important to note that some studies have observed a concomitant presence of CECS and functional PAES in many patients [5]. It is for this reason that presence of early evidence supporting CECS should not lead the clinician into forgoing investigation of vascular causes of pathology. Our patient did in fact have elevated compartment pressure in the superficial and deep posterior compartments; however compartment pressures normally rise to some extent during exercise and his anterior compartment was not affected [18]. These pressures were likely misleading, as CECS alone is not associated with a dynamic distal vascular exam.

Medial tibial stress syndrome (MTSS), commonly referred to as shin splints, and tibial stress fractures are another common cause of leg pain in athletes and are of particular concern in military populations [27]. Physical exam typically reveals tenderness about the middle to distal one-third of the tibia. Examination of the ankle and neurovascular status is normal. Radiographs may also be normal in this condition but bone scan will likely be positive [28]. While these diseases often present with similar findings, appropriate clinical examination to include timing of onset and radiographic studies should help in the differentiation. Our patient's physical examination and radiographic work-up yielded none of the bony pathology consistent with medial tibia stress syndrome or stress fracture and are mentioned here for completeness of our differential diagnosis.

Proximal compression of the tibial nerve as it passes through the origin of the soleus is yet another cause of posterior leg pain that may confound the diagnosis. Williams comments that much of the proceeding literature focused on the above diagnoses may have failed to evaluate the role of the soleus in causing neuropathic pain described by patients in the studies [22]. Williams postulates that some of the deep compartment syndrome patients may have in fact had only tibial nerve compression and that fasciotomies were relieving the pain by opening the soleal sling and releasing the proximal tibial nerve rather than relieving compartmental pressures. Gentle palpation over the posterior midline of the distal popliteal fossa where the tibial neurovascular bundle passes under the soleus should produce pain out of proportion to exam in patients with soleal sling syndrome [24]. Also isolated flexor hallucis longus weakness in conjunction with posterior leg pain may be indicative of soleal sling compression. Furthermore, the author suggests that electrodiagnostic testing and magnetic resonance imaging were neither sensitive nor specific for this syndrome. It was noted that EMG was beneficial in patients with possible confounding lumbar disk disease, and MR was helpful in ruling out other compressive masses such as gangliomas and occasionally helped when the soleal sling was particularly fibrous [25, 29]. Other, more contemporary studies have shown the benefit of MR in the diagnosis. A study done by Ladak reliably found a thickened soleus sling with T2 enhancement of the tibial nerve at the level of the sling and was able to elucidate denervation changes in muscles of the posterior compartment of the leg thereby demonstrating etiology [26].

In summary, PAES is a rare but significant cause of leg pain in the athletic population. Because the diagnosis of PAES relies heavily on a careful vascular exam, this entity is more recognized in the Vascular Surgery literature. Other items on the differential diagnosis include chronic exertional compartment syndrome, medial tibial stress syndrome, soleal sling syndrome, and tibial stress fractures, all of which are more common in most orthopaedic clinics (Table 2). However, patients with PAES do present to orthopaedic and sports medicine offices and the diagnosis of PAES should not be overlooked as its missed diagnosis can result in delays in treatment, the potential morbidity of the wrong surgical procedure, and potential for serious sequelae as arterial damage progresses. We attempt to delineate a suggested work-up in patients with exertional calf pain of uncertain etiology. While this attempt is not proven empirically, its formulation through review of literature makes it a good starting point for clinicians encountering a confusing patient.

## Figures and Tables

**Table 1:** Pre- and postexercise compartment pressure measurements in the left lower extremity.

Compartments	Left lower extremity compartment pressure measurements (mmHg)	
	Preexercise	Postexercise
Anterior	42	48
Lateral	53	58
Superficial posterior	31	50
Deep posterior	34	66

**Table 2:** Key features distinguishing common sources of leg pain in the athlete.

	Chronic exertional compartment syndrome	Medial tibial stress syndrome	Tibial stress fracture	Soleal sling syndrome	Popliteal artery entrapment syndrome
Primary site of pathology or structure(s) affected	Fascial compartments^a^ [19]: Anterior (45%) Deep posterior (40%) Lateral (10%) Superficial posterior (5%)	Distal posteromedial 1/3 of tibial shaft [8, 20, 21]	Proximal tibial metaphysis or diaphysis [8, 20, 21] Mid-diaphysis more common in runners [8]	Tibial nerve as it passes through origin of soleus [8, 22]	Anatomic: aberrant anatomy of proximal gastrocnemius, popliteal artery, or both [1, 8] Functional: hypertrophied proximal gastrocnemius compresses artery during exercise [2, 8]

Key identifying symptom(s)	Diffuse painful cramping, burning, “fullness” in leg [8, 20] Paresthesias with exertion [8, 20]	Recurrent localized, dull, bony ache [8]	Insidious onset localized leg pain Classically improving mid exercise and then returning at end of exercise [8]	Pain or paresthesias in nerve distribution worse with exertion	Exertional calf pain, cramping, tensing, and claudication symptoms Paresthesias in sole of the foot (tibial nerve) [5]

Key identifiers from patient history	Recurrent with exertion Running and jumping type activities [8, 20, 23] Bilateral (85 to 95% of cases) [19]	Often late in sports season or periods of increased training intensity [8]	History of eating disorder, female athlete triad^b^, repetitive high-impact activities (marching, running, jumping) [8]	Pain with activity, worse with continued activity [8]	Predominantly males under thirty years old [8] High-intensity exercise with significant PF and DF at the ankle

Key finding(s) of physical exam	Compartment tenderness and tensing in immediate postexercise period [8] Pain with passive stretch of affected muscles in immediate postexercise period [8, 23]	Palpable bony tenderness over medial border of distal tibia [8, 19, 20]	Localized, bony tenderness to palpation over fracture site [8] Vibratory pain from tuning fork [8]	Pain out of proportion with palpation over posterior midline of distal popliteal fossa [24] Positive Tinel sign at site of nerve compression [8] Isolated FHL weakness [24]	Weaker distal pulses compared to uninvolved side, or attenuation of pulses with foot positioned in DF or PF with knee extension [5–8]

Diagnostic modalities of choice	Intracompartmental pressure (ICP) measurements continuously during exercise [23] more reliable than pre- and postexercise [8]	Radiographs triphasic bone scan if radiographs negative	Radiographs triphasic bone scan if radiographs negative	Diagnostic nerve block [8] EMG rule out confounding lumbar disc disease [25] T2 MRI enhancement showing thickened soleus sling [26]	Provocative ABI with ankle PF or DF [5–7] Dynamic CDUS Dynamic MRI/MRA or CTA [8–11, 14] Arteriography is gold standard [8]

^a^Compartments: anterior: deep peroneal nerve. Deep posterior: tibial nerve. Superficial posterior: sural nerve. Lateral: superficial peroneal nerve. ^b^Female athlete triad: eating disorder, amenorrhea, and osteoporosis. FHL: flexor hallucis longus, PF: plantarflexion, DF: dorsiflexion, and ABI: ankle-brachial index.
